# Metastatic pulmonary artery sarcoma presented with tamponade: a case report

**DOI:** 10.1002/ccr3.989

**Published:** 2017-05-10

**Authors:** Shokoufeh Hajsadeghi, Siavash Kooranifar, Nafise Ansarinejad, Alireza Sadeghipour, Aida Iranpour, Alireza Aziz Ahari

**Affiliations:** ^1^ Department of Cardiovascular DiseaseHazrat‐e Rasool General HospitalIran University of Medical SciencesTehranIran; ^2^ Department of Pulmonary and Critical CareHazrat‐e Rasool General HospitalIran University of Medical SciencesTehranIran; ^3^ Department of Hematology and OncologyHazrat‐e Rasool General HospitalIran University of Medical SciencesTehranIran; ^4^ Department of PathologyHazrat‐e Rasool General HospitalIran University of Medical SciencesTehranIran; ^5^ Department of Internal MedicineHazrat‐e Rasool General HospitalIran University of Medical SciencesTehranIran; ^6^ Department of RadiologyHazrat‐e Rasool General HospitalIran University of Medical SciencesTehranIran

**Keywords:** Positron emission tomography, single‐nucleotide polymorphism, transthoracic echocardiography

## Abstract

Pulmonary artery sarcoma is a rare tumor with varying presentations including pericardial effusion and pulmonary metastasis. Single‐nucleotide polymorphism array is a novel method that can be used to define tumor genome and be used as a guidance to choose the proper treatment regimen.

## Introduction

Pulmonary artery sarcomas (PAS) are rare tumors with varying presentations including pulmonary artery obstruction, pulmonary hypertension, and intrathoracic or extrathoracic metastasis 1.Metastatic pulmonary sarcomas are even more uncommon, and to our knowledge, have only been reported in one case 2. We report this case to highlight a novel presentation of this tumor and to discuss the challenge in using different treatment modalities.

## Case Presentation

**Figure 1:**
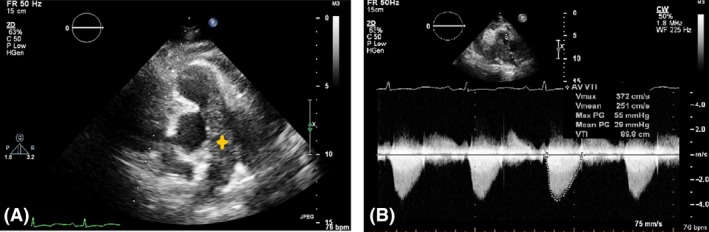
Panel (A) Parasternal short‐axis view of major arteries in TTE. A large, lobulated intravascular mass extending from the vicinity of the PV into the RPA with partial occlusion of the lumen is showed. Panel (B) Continuous‐wave CW Doppler of the pulmonary artery. A significant peak pressure gradient (PPG: 55 mmHg), suggestive of partial occlusion of the main PA is showed. PA, pulmonary artery; RPA, right pulmonary artery; PV, pulmonary valve.

**Figure 2:**
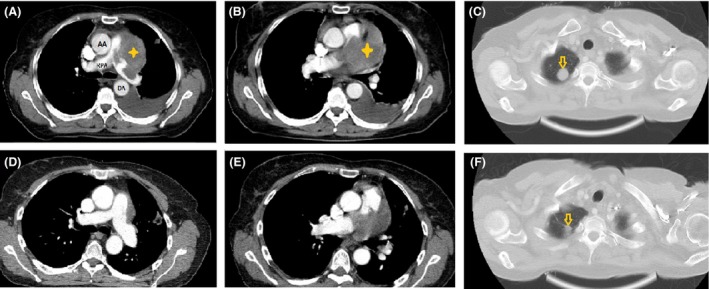
Intravenous contrast‐enhanced CT scan images of patient's thorax. First row images are taken at first presentation and second row images are related to the last CT scan after 9 months of chemotherapy. (A, B) (in mediastinal window) show a left mediastinal mass (which invades the main pulmonary trunk and left main pulmonary artery. After treatment, CT scan (D, E) showed more than 30% size reduction of the mass, suggestive of partial response. (C) (lung window) shows a metastatic nodule (yellow arrow) in pulmonary left upper lobe which becomes faint and smaller (F) after treatment. AA, ascending aorta; RPA, right pulmonary artery; DA, descending aorta.

A 69‐year‐old woman was hospitalized because of palpitation, nausea, and vomiting. She reported a history of dyspnea and pleuritic chest pain one month prior to hospitalization that was gradually worsened. Her past medical history included hypertension and chronic kidney disease. The physical examination showed several remarkable elements, including hypoxemia at room air, decreased blood pressure, and muffling of heart sounds. Because cardiac tamponade was our first concern in the diagnosis, bedside transthoracic echocardiography (TTE) was performed, which revealed moderate pericardial effusion with tamponade physiology. Patient underwent emergent pericardiocentesis and 800 cc of bloody fluid was drained. TTE was also revealing a large lesion in the main pulmonary artery, with extensions into the right and left pulmonary arteries (Fig. 1). In order to determine the exact burden of the lesion, contrast‐enhanced computed tomography (CT) was performed, and showed that the lesion was predominantly in the main and left pulmonary arteries, and extended into the left mediastinum, which was associated with a pulmonary nodule in the right upper lobe(Fig. 2, first row).

**Figure 3:**
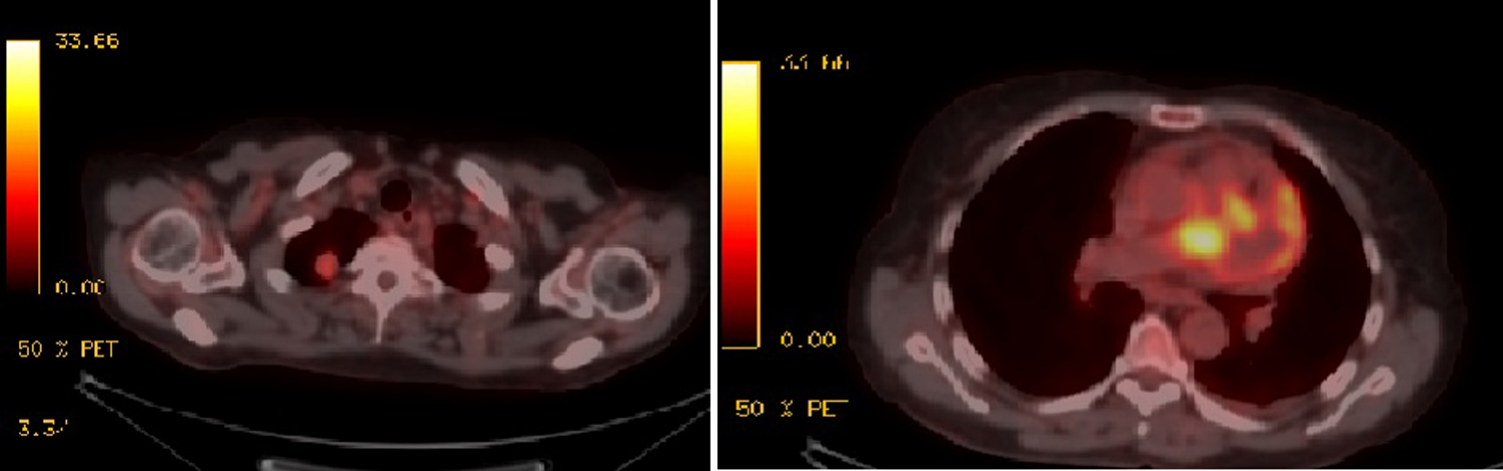
PET‐CT revealed an increased 18‐FDG‐standardized uptake value (SUV) of 4.0 in the right upper lobe nodule (left), associated with increased uptake of a soft tissue density mass in the perivascular space of the anterior mediastinum with a max SUV 10.9 (right).

Analysis of pericardial effusion showed lymphocyte‐predominant fluid with negative cytology. Subsequently, 18‐fluorodeoxyglucose (18‐FDG) positron emission tomography–CT (PET‐CT) from the vertex to the thighs was performed and revealed an increased 18‐FDG‐standardized uptake value in the right upper lobe nodule and in a soft tissue density mass in the perivascular space in the anterior mediastinum (Fig. 3).

**Figure 4:**
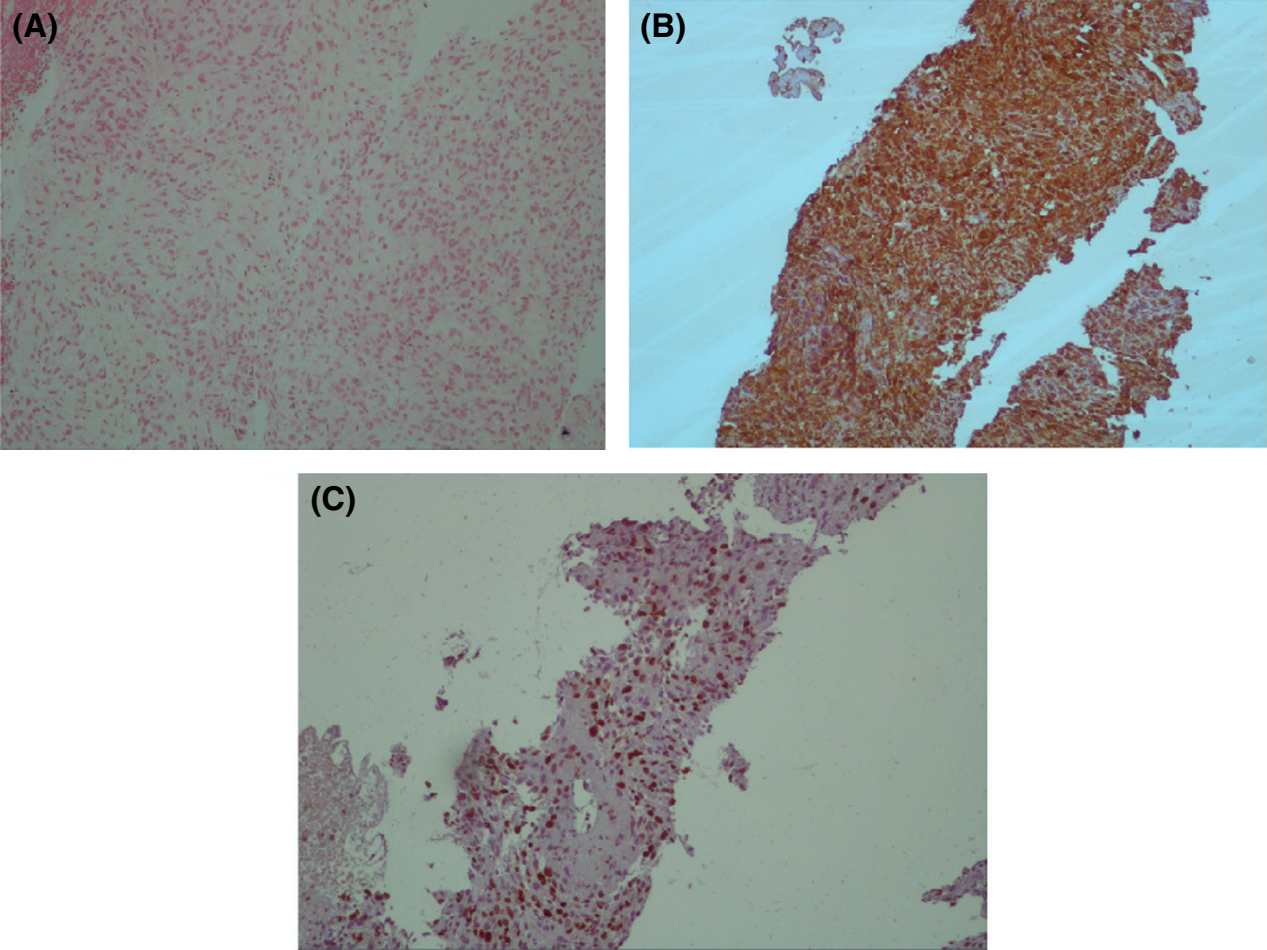
Panel (A) Short twisted bundles of elongated to plump spindle mesenchymal cells in a myxoid stroma (H&E, 250×). Panel (B) Diffuse cytoplasmic reactivity of neoplastic cells for vimentin (monoclonal mouse anti‐vimentin clone V9, ×100). Panel (C) High proliferation activity of tumor cells demonstrated by Ki67 staining (monoclonal mouse anti‐human Ki67 clone; MIB‐1, 100×).

The patient received antibiotics for diagnosis of pneumonia and a CT‐guided biopsy of right parenchymal nodule was performed. A pathologic examination showed neoplastic tissue composed of pleomorphic cells, while immunohistochemistry of the tumor cells was positive for vimentin and negative for Pan CK, EMA, S‐100, HMB‐45, SM‐actin, desmin, CD 31, CD 34, and CD 68, while 30% of tumor cells showed reactivity to Ki67. All findings were suggestive of high‐grade pleomorphic sarcoma (Fig. 4). Three courses of chemotherapy consisting of gemcitabine 900 mg/m^2^ (days 1 and 8) and docetaxel 100 mg/m^2^ (day 8) with 21 days’ interval were prescribed. After 3 months, TTE and CT scans were repeated, but unfortunately, we found no improvement. At this time, a single‐nucleotide polymorphism (SNP) analysis of tumor's genome was planned. Venous blood sample of 5 mL has been collected to EDTA‐covered tube and the DNA was extracted. The analysis of rs2032582 (SNP) in tandem repeats of ABCB1 gene was carried out using allele‐specific polymerase chain reaction. This test revealed tumor susceptibility to doxorubicin. Based on these findings, a new chemotherapy regimen was started, containing ifosfamide 5000 mg/m^2^ (day 1) and Adriamycin 75 mg/m^2^ (day 1) with 21 days’ interval. Patient received four course of chemotherapy and subsequent CT scans following the end of the forth course of chemotherapy revealed partial response to chemotherapy and partial resolution of the pulmonary nodule and pulmonary artery mass (Fig. 2, second row). The patient has thus far survived eleven months and currently is receiving chemotherapy.

## Discussion

Pulmonary artery sarcomas are rare tumors that the literature suggests are often mistaken for pulmonary artery thrombi 3. They contain some radiologic features that suggest malignancies rather than thrombi, such as the distention of vascular lumen, invasion of adjacent tissue, heterogeneous appearance, and evidence of distant metastasis 4. In our patient, because the lesion seemed to invade the mediastinum and was associated with a right pulmonary nodule, a malignant lesion arising from the pulmonary artery was more probable than a thrombus. Among different radiologic imaging modalities, PET has high sensitivity and high negative predictive values, making it a very useful tool for assessing lung neoplasm 5. In our patient, tissue biopsy of the mediastinal mass was associated with great harm, so we performed 18 FDG PET‐CT from the vertex to proximal thighs to determine whether a biopsy of pulmonary mass would be helpful and assist us in locating distant metastasis.

Although surgical resection is the standard treatment for pulmonary artery sarcoma, these tumors usually present as late, bilateral, locally advanced, and/or metastatic, which decrease the possibility of successful operation 6.According to the proposed staging system for primary pulmonary artery sarcoma, our patient was classified as stage 2, denoting involvement of the main pulmonary artery plus one lung, so she was a candidate for receiving chemotherapy 7. Although different chemotherapy regimens have been reported to be effective for pulmonary artery sarcoma, anthracyclines, either alone or in combination, are the most commonly used agents 7, 8. SNP arrays have become a key technology for analysis of the cancer genome to define new disease loci and guide therapeutic decisions 9. After the first course of chemotherapy was failed, our patient was candidate for receiving Adriamycin, but cardiovascular complications were our major concern so a SNP analysis was performed. When tumor susceptibility to Adriamycin was proved, a second treatment course containing Adriamycin was started. Because pericardial effusion and metastasis are not common presentations of PAS, and different treatment recommendations exist for this condition, we decided to report the above case.

## Authorship

SH and SK: wrote the draft of the manuscript and obtained the consent. NA: performed data interpreting regarding to SNP analysis and participated in the writing. AS, AI, and AA: performed literature review and language editing and gave final approval of the manuscript. All authors have read and approved the final manuscript.

## Conflict of Interests

The authors declare that they have no competing interests.

## References

[1] Dias, O. M. , E. M. Lombardi , M. Canzian , J. SoaresJúnior , L. D. Vieira , and M. Terra Filho . 2011 18F‐fluorodeoxyglucose positron emission tomography as a noninvasive method for the diagnosis of primary pulmonary artery sarcoma. J. Bras. Pneumol. 37:817–822. 22241041 10.1590/s1806-37132011000600017

[2] Medalie, N. S. , C. E. Vallejo , and P. Wasserman . 1998 Metastatic pulmonary artery sarcoma. Acta Cytol. 42:968–972. 9684587 10.1159/000331978

[3] Nakamura, Y. , T. Shimizu , Y. Fukumoto , K. Sugimura , S. Ito , F. Fujishima , et al. 2012 A case of angiosarcoma arising in trunk of the right pulmonary artery clinically simulating pulmonary thromboembolism. World J. Oncol. 3:119–123. 10.4021/wjon467w PMC5649790 29147292

[4] Kim, J. H. , F. R. Gutierrez , E. Y. Lee , J. Semenkovich , K. T. Bae , and L. R. Ylagan . 2003 Primary leiomyosarcoma of the pulmonary artery: a diagnostic dilemma. Clin. Imaging 27:206–211. 12727062 10.1016/s0899-7071(02)00568-5

[5] Kostakoglu, L. , H. Agress, Jr. , and S. J. Goldsmith . 2003 Clinical role of FDG PET in evaluation of cancer patients 1. Radiographics 23:315–340. 12640150 10.1148/rg.232025705

[6] Wong, H. H. , I. Gounaris , A. McCormack , M. Berman , D. Davidson , G. Horan , et al. 2015 Presentation and management of pulmonary artery sarcoma. Clin. Sarcoma Res. 5:1. 10.1186/s13569-014-0019-2 PMC4307142 25628857

[7] Blackmon, S. H. , D. C. Rice , A. M. Correa , R. Mehran , J. B. Putnam , W. R. Smythe , et al. 2009 Management of primary pulmonary artery sarcomas. Ann. Thorac. Surg. 87:977–984. 19231448 10.1016/j.athoracsur.2008.08.018

[8] Nakahira, A. , H. Ogino , H. Sasaki , and N. Katakami . 2007 Long‐term survival of a pulmonary artery sarcoma produced by aggressive surgical resection and adjuvant chemoradiotherapy. Eur. J. Cardiothorac. Surg. 32:388–390. 17566754 10.1016/j.ejcts.2007.04.019

[9] Heinrichs, S. , C. Li , and A. T. Look . 2010 SNP array analysis in hematologic malignancies: avoiding false discoveries. Blood 115:4157–4161. 20304806 10.1182/blood-2009-11-203182 PMC2879098

